# Changes in Natural Silk Fibres by Hydration, Tensile Loading and Heating as Studied by ^1^H NMR: Anisotropy in NMR Relaxation Times

**DOI:** 10.3390/polym14173665

**Published:** 2022-09-03

**Authors:** Victor V. Rodin, Peter S. Belton

**Affiliations:** 1 Institute of Organic Chemistry, Johannes Kepler University Linz, Altenbergerstraße 69, 4040 Linz, Austria; 2 School of Chemistry, University of East Anglia, Norwich NR4 7TJ, UK

**Keywords:** water anisotropy, polymer, *B. mori* silk, ^1^H NMR relaxation, *T*_1_, *T*_2_, double quantum filter (DQF) NMR

## Abstract

*B. mori* silkworm natural silk is a fibrous biopolymer with a block copolymer design containing both hydrophobic and hydrophilic regions. Using ^1^H NMR relaxation, this work studied *B. mori* natural silk fibres oriented at 0° and 90° to the static magnetic field *B*_0_ to clarify how measured NMR parameters reflect the structure and anisotropic properties of hydrated silk fibres. The FTIR method was applied to monitor the changes in the silk I and *β*-sheet conformations. Unloaded *B. mori* silk fibres at different hydration levels (HL), the silk threads before and after tensile loading in water, and fibres after a stepped increase in temperature have been explored. NMR data discovered two components in *T*_1_ and *T*_2_ relaxations for both orientations of silk fibres (0° and 90°). For the slower *T*_2_ component, the results showed an obvious anisotropic effect with higher relaxation times for the silk fibres oriented at 90° to *B*_0_. The *T*_1_ component (water protons, HL = 0.11) was sequentially decreased over a range of fibres: 0° oriented, randomly oriented, silk *B. mori* cocoon, 90° oriented. The degree of anisotropy in *T*_2_ relaxation was decreasing with increasing HL. The *T*_2_ in silk threads oriented at 0° and 90° also showed anisotropy in increased HL (to 0.42 g H_2_O/g dry matter), at tensile loading, and at an increasing temperature towards 320 K. The changes in NMR parameters and different relaxation mechanisms affecting water molecular interactions and silk properties have been discussed. The findings provide new insights relating to the water anisotropy in hydrated *Bombyx mori* silk fibres at tensile loading and under a changing HL and temperature.

## 1. Introduction

To repair tissues or organs in the body, natural polymers taken from animal or plant world are often used as materials because of their properties [1,2]. Such natural materials are applied in fabricating scaffolds for prospective biomedical engineering [2,3,4,5]. These materials should possess excellent physical-mechanical properties such as replaced tissue. In addition to these, the material should be non-toxic, easy to produce and available. Silks are natural biological composite materials with attractive properties for these applications [2,3,4]. People already use silk proteins for textile production [1,3,5]. Silk is also regarded as a non-degradable biomaterial applied to construct biomedical textiles [4,5,6]. After several months of implantation in vivo, silk fibroin (SF) fibres retain more than 50% of their original mechanical properties [1,5].

Silk is a protein polymer of amino acids. The amino acids are linked by amide bonds, resulting in typical polyamide [3]. Two main silk proteins are fibroin and sericin [1,2,3,4]. Moreover, fibroin is the structural core/centre of raw silk fibre, whereas sericin is the sticky protein covering the silk core. The sequence of amino acid residues is a primary structure of silk. The primary structure of silk is mostly presented by the amino acids of glycine (Gly), alanine (Ala), and serine (Ser). Fibroin is composed of the amino acids Gly-Ser-Gly-Ala-Gly-Ala, i.e., Gly and Ala are mostly found in fibroin, whereas Ser is the main amino acid residue in sericin. These residues are connected in a specific repeating pattern. Intrachain and interchain H-bonding (mostly hydrogen bonding between chains) causes the polymer chains to have a zigzag conformation. This is the secondary or *β*-sheet structure of silk polymers [3,4,5].

The range of silk protein properties that are useful in many applications has permanently become wider: silk-based materials have applications in regenerative medicine, tissue engineering, drug delivery, and pharmacy [2,3,4,5,7]. *Bombyx mori* silk, produced by silkworm, is considered one such natural biomaterial [1,3,6,8].

Three main residues (Gly), (Ala), and (Ser) in *B. mori* silk contain short side-chains and permit close packing through the stacking of H-bonded *β*-sheets. Studies on the structure of *B. mori* silk fibroin (SF) have revealed a number of conformational states; however there is controversy concerning the different data [3,4,5,8,9,10]. It is generally accepted that *B. mori* silk fibroin has three major conformations in the solid state, which are random coil, silk I structure and *β*-sheet (or silk II structure) [3,4,5,6,7,8,9].

The materials from *Bombyx mori* silk, SF properties and water dynamics in silk fibres have been studied using different methods, such as Fourier transform infrared (FTIR) spectroscopy, differential scanning calorimetry (DSC), thermo-gravimetric analysis (TGA), X-ray diffraction (XRD), electronic microscopy, Raman spectroscopy, ^13^C CP/MAS and DD/MAS NMR, ^1^H and ^2^H NMR relaxations, pulsed field gradient NMR (PFG) and 2D NMR correlations, ^1^H double-quantum-filtered (DQF) NMR and MRI [6,7,8,9,10,11,12,13,14,15,16,17,18,19,20,21,22,23]. For instance, the work [10] showed that ^2^H NMR is sensitive to fast water motions and an exchange in SF fibres and films interacting with water. In that study, SF film samples were placed in an NMR tube with ^2^H_2_O for one day or more, and then (^2^H) relaxation of water was measured. For the Ala-, Ser- and Tyr- residues, the conformations were determined with ^13^C DD/MAS NMR, and compared with the conformations obtained for the hydrated SF fibres [10]. According to publications [10,17,20], Ala- residues are presented in both domains (the crystalline and non-crystalline domains) whereas Tyr residues are mostly found in the non-crystalline domains of the silk samples. Ser- residues are mainly found in the crystalline domains. Asakura et al. [10] applied ^2^H *T*_1_–*T*_2_ and *T*_2_–*T*_2_ NMR methods for studying the interactions between water and SF fibres and films showing that the clarification of the exchange conditions in silk materials can be achieved with the aid of a two-dimensional inverse Laplace transform. This approach identified different types of water on 2D maps.

The mechanical properties of silk-based materials are determined by the arrangement of their secondary structure [1,2,4,5]. This structure also defines how silks interact with water [6,10,12,13,14,22,24,25,26]. The data considered in publications [6,22] provided the details of how the hydration of the silk films affects their thermal stability. When SF films were kept in saline solution, a decrease in the modulus of Young and increase in plastic deformation were discovered [6]. The content of *β*-sheets increased in water-annealed silk films when water content was increasing [6]. In the silks, hydrophobic domains are composed from amino acids (the primary sequence) with short side-chains. The *β*-sheet structures are formed due to these hydrophobic domains as part of the packing of stacked sheets of anti-parallel chains that are connected by H-bonds in protein [2,3,22]. There are smaller hydrophilic chains among these large hydrophobic domains. They form hydrophilic links between hydrophobic domains using polar side chains. The silk II structure (*β*-sheets) fosters the stability of silk fibres in the protein and contributes to the strength of silk. The elasticity of the silk fibres is due to the silk I structure [22]. The hydrophilic domains (polar side chains) results in the formation of the amorphous part of the secondary structure [2]. As a result of the combination of hydrophobic and hydrophilic domains in the silks produced from silkworms, the SF fibres *Bombyx mori* possess attractive mechanical characteristics for Young’s modulus, ultimate tensile strength, environmental stability, and morphological flexibility [1,5,22]. In the *β*-sheet structure, the methyl groups and hydrogen groups of opposing sheets interact to form intersheet stacking. Strong H-bonds and van der Waals forces generate a stable structure [4,22]. Despite the stable *β*-sheet structure in the silk fibres, water can affect different properties of the fibres, starting with the influence on hydrophilic places [25,26]. The publications [10,11,12,13,14] also demonstrated that mechanical properties of silk-based materials depended on water or saline solutions after immersing silk materials in solution. The changes in the preliminary history of extracting silk fibres and environmental medium (hydration, temperature, presence of additives), as well as shear stress, can result in conformation transitions and changes in strength, toughness and stiffness [1,5,9,13,15,19,21]. The maximal stress σ_max_ in *B. mori* silkworm silk fibres decreased when a diameter of fibre increased or more heating silk was applied [1,13,14,15,16,21,22,23]. In the DSC and TGA studies of silk films [22,23], the authors considered that the plasticizing effect of bound water results in a lower temperature for the glass transition of the silk. With the increasing temperature in hydrated silk films, the loss of bound water has a place. The silk fibroin loses all bound water when the temperature is higher than it is for the lower temperature glass transition. Then, with this reduced mobility of the molecules in the dried silk, the higher-temperature glass transition becomes observable [22]. Mechanical treatment, temperature changes and solvents can result in a stable *β*-sheet structure, i.e., silk II conformation [22,23,26]. The effect of water on the *T*g of material at moisturising was observable as the plasticization of fibroin film [25,26]. The work [24] studied the effects of salts on the silk phase behaviour and showed that, with increasing osmotic pressure, the structure of silk I in silk fibroin converts into an antiparallel β-sheet structure, silk II. The temperature treatment of the *B. mori* silk fibres changed the strain *ε* (relative elongation) and maximal stress σ_max_ [13,15,16]. MRI monitored the changes in the water state of hydrated *B. mori* silk fibres by recording images of cross-section and longitudinal slices [14].

Plaza et al. [13] discussed a contraction phenomenon for unrestricted silk fibres when the fibres were immersed in water. It is very important to understand the hydration process of proteins in natural silk and silk-based materials and the role of the non-crystalline domains in the interaction of water with silk fibres [10,12,13,14]. This results in disruption of the hydrogen bonding in these regions by the strong interaction of water molecules with polar groups of the SF chain. However, at the molecular level, the hydration mechanism of water interaction with SF has not been completely clarified [13,14,15,19,20,21,22,24,25,26].

The toughness and strength of silks produced by silkworms grew if the sericin content in the fibres was decreased. Also, at a decreasing fibre diameter, the growth in the toughness and strength of silk had a place [1,14]. The same tendency for toughness and strength was observed upon an increase in the secondary structure content [8]. In the work [10], the authors presented how CP/MAS NMR studied various heterogeneous structures of SF *B. mori* generated by different stretching lengths upon hydration. In the works [8,10,17], CP/MAS NMR and ^2^H NMR were applied to investigate the dynamics of molecules at water-SF interactions. The tensile strength and elasticity of SF *Bombyx mori* were dependent at the water sorption level [10,25,26].

Subsequent applications of natural polymers such as silk *B. mori* strongly depend on understanding the effects of water and temperature on the silk proteins [5,22,23,24,25,26]. In the works [17,20], the solid-state NMR data on a number of different silks in hydrated states were discussed. NMR studies showed that some amino acids moved isotropically [8]. Questions on the isotropic-anisotropic mobility of water and transitions in water states in various natural fibrous materials and tissues such as collagen, tendon, cartilage, muscle, and nerves were studied using different NMR methods [16,19,27,28,29,30,31,32,33,34,35,36,37]. In highly organized tissues, the measurable NMR parameters are dependent on the orientation of the tissue constituents with respect to the magnetic field *B*_0_ [27,28,29,30,31,32,33]. These NMR methods seemed to be very informative in detailing the anisotropic properties of biological tissues when measuring *T*_1_, *T*_2_, apparent diffusion coefficient (ADC) [29,30,31,32,33,34,35]. For instance, the findings of Takamiya et al. [30] showed the bi-exponential *T*_2_ relaxation of water in tendon (Japanese white rabbit) oriented at angles of 0, 35, 54.7, 75, and 90° to the static magnetic field and anisotropy in both the short *T*_2_ and long *T*_2_ components.

The anisotropy of water connected with collagen at fibre orientations in sheep tendon in the magnetic field was explored using ^1^H DQF NMR signals [34]. The DQF NMR method based on sensitivity to residual dipole-dipole interactions in water trapped in anisotropic environments was also applied to the study of randomly oriented *B. mori* silk fibres [14,16,19]. However, this technique has not been used to explore natural silk fibres with a defined orientation to the static magnetic field. When studying the ADC in Achilles tendon with varying orientations to static magnetic field [28,33], the self-diffusion anisotropy of water was discovered. The PFG NMR technique has been used to explore ADC of water in randomly oriented natural silk fibres *B. mori* [12,14], but not in the silk treads with a defined orientation to the static magnetic field. For oriented hydrated collagen fibres [32], the ADC of water was shown to depend on cross-link level and is different for two directions of applied gradients (at 0° and 90° to the field *B*_0_). The works [29,38] studying the anisotropic characteristics of *T*_2_ multi-components and *T*_1ρ_ relaxation times in tendon have shown *T*_2_ and *T*_1ρ_ changes at different tendon orientations. According to NMR studies on oriented collagen tissues [28,29,30,32,34,35,36,39], the alignment of the bound water follows collagen fibres. This effect is based on the orientation anisotropy observed by *T*_2_ relaxation/ADC behaviour. To date, the *T*_2_ relaxation of water protons in natural silk *B. mori* at different fibre orientations in magnetic field has not been studied.

In publications [31,35], 2D NMR correlation spectroscopy was applied to study the distribution of diffusion coefficients in two orthogonal directions in anisotropic systems, showing how diffusion coefficients in two orthogonal directions (at 0° and 90°) in fibres can be measured in the experiments using the collinear and orthogonal orientations of applied gradients. Using this approach, they could be reflected by 2D correlation maps. However, anisotropy-connected fibre orientation in silk *B. mori* samples has not been explored using ^1^H-developed NMR approaches.

The current work investigates how orientational anisotropy in natural silk fibres *Bombyx mori* (oriented at 0° and 90° to the *B*_0_) at varying hydration levels, increasing temperature and mechanical loading is reflected in measurable NMR relaxation parameters. This NMR work results in findings regarding the dynamical properties of water interacting with silk fibres *Bombyx mori* in conditions when their anisotropic properties could be observed. Additionally, FTIR spectroscopy was applied to explore the effects of hydration, heating and tensile loading on the conformational characteristics of natural silk *B. mori*. The FTIR method highlighted the changes in the bands of the spectra responsible for silk I and *β*-sheet structures. The NMR findings for unloaded silk fibres at different hydration levels, for the silk threads before and after tensile loading wet fibres, and for the silk samples after stepping increase in the temperature were obtained. NMR data discovered two components in *T*_1_ and *T*_2_ relaxations for both orientations of silk fibers (in parallel and perpendicular to field *B*_0_). For the slower *T*_2_ components, the results showed an obvious anisotropic effect, and the degree of anisotropy decreased with the increase in hydration level. The *T*_2_ values for both parallel and perpendicular orientations of silk threads to *B*_0_ also showed anisotropy at increased humidity levels (HL) of up to 0.42 g H_2_O/g dry matter, at tensile loading wet fibres, and at slow heating to 320 K. Some relaxation mechanisms that affect molecular interactions in hydrated silk fibres and NMR parameters were analysed. The NMR data provide new insight relating the water anisotropy in hydrated silk fibres *Bombyx mori* at tensile loading, increasing temperature and changing water content.

## 2. Experimental Materials and Methods

### 2.1. Samples

The natural silk fibres and the samples of silkworm *B. mori* cocoon were obtained from Oxford Biomaterials, Ltd. (Oxford, UK). In NMR experiments, randomly distributed *B. mori* threads or oriented fibres were placed on the bottom of 5 mm Wilmad NMR tubes. (SP Industries, Inc.; Warminster, PA, USA). In silk samples prepared for NMR studies of anisotropy, the silk threads with a length of roughly 1 cm (to fit the measurable size within the coil) were aligned in parallel orientation to each other and along the axis of tube. To prepare the samples with the perpendicular orientation of fibres to tube axis, the silk treads were spirally wound around a glass rod. Then, the glass rods with wound silk threads were inserted into NMR tubes. The tubes were firmly closed with Teflon caps and PTFE tape. Separately, empty NMR tubes with cap, rod and PTFE tape have been checked for any residual NMR signal to subtract this (if it exists) from the main measurement with silk fibres.

### 2.2. Mechanical Loading Tests

#### 2.2.1. Deformation Measurements on the Normal Silk Fibres

**Figure 1:**
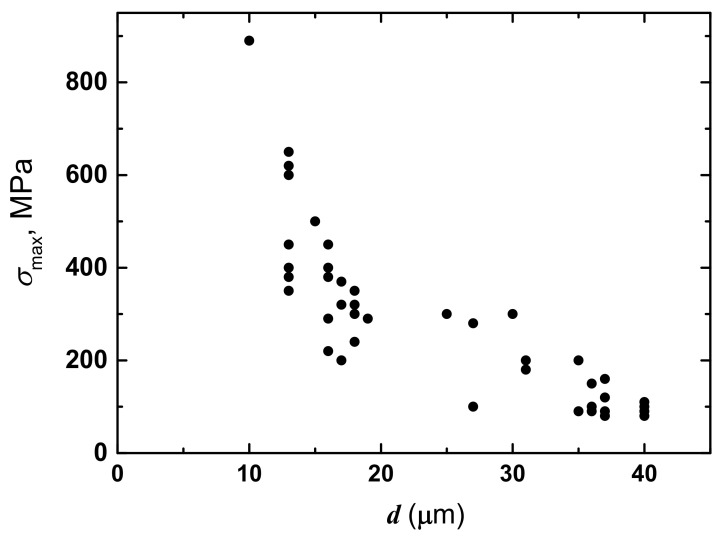
Dependence of the maximum stress σ_max_ in linear elastic region on the diameter *d* of the initial natural silk threads.

In the initial experiments with mechanical loads, the silk fibres *B. mori* were explored without any preliminary treatment, i.e., individual silk threads were tested for longitudinal tension under increasing load *p* from 0 to 1 N with INSTRON universal testing machine (INSTRON, Buckinghamshire, UK), as was described before in the publications [14,15]. The elongation of sample Δ*L* as a function of load was recorded with recorder at chart rate of 100 mm/min (with traverse velocity of 2 mm/min). With relative elongation Δ*L*/*L*_0_, the strain *ε* could be calculated as *ε* = (Δ*L*/*L*_0_) × 100%. These mechanical tests produced tension diagrams in (Δ*L-p*) coordinates according to the concepts of material mechanics [15,40]. Then, stress σ was calculating by dividing the load *P* by the initial cross-sectional area of the thread. Thus, the data were converted into σ-*ε* diagram, showing that, at low loads, Hooke’s law was valid [40]. Some silk fibres were heated at 50° C and then measured with loads again. The deformation measurements of the initial and heat-treated states were carried out at room temperature. The values of *σ*_max_ and ε_max_ were compared for initial and treated fibres (the maximal stress and maximal strain as elastic limit in a linear region where Hooke’s law is valid according to *σ* = *E* × ε, where *E* is Young’s modulus [40]). Figure 1 shows some deformation characteristics *σ*_max_ of the threads of variable diameters. *ε*_max_ had no clear dependence on fibre diameter, whereas σ_max_ decreased with an increase in silk fibre diameter.

The large-diameter fibres differ from small-diameter ones by the increased number of intermolecular cross-links. With this decrease in σ_max_, there is a narrowing of the strain range where Hooke’s law for elastic deformation is valid. These findings are in line with the previous publications [14,15,20].

After exposing silk fibres to heat, σ_max_ diminished, and this decreased with increasing fibre diameter demonstrating an impairment of the strength characteristics of threads. An approximately twofold decrease in the slope of liner part in the diagram of σ-*ε* was discovered for heated fibres. The data could be used to group further silk fibres with a more narrow range of diameters for NMR studies of anisotropy.

#### 2.2.2. Tensile Tension of Silk Threads with Constant Mechanical Loads

The effects of tensile loading on the NMR parameters (*T*_1_ and *T*_2_) and the amide I and amide II regions in the FTIR spectra of silk were measured as follows. The load with mass 89 g or the one with 135 g (to create different elongation) was applied to silk threads in gravity field to produce constant longitudinal tensile tension (for 6–12 h). In mechanical loading studies, the silk *B. mori* threads with the length *L*_0_ = 330 mm and *L*_0_ = 247 mm were tested, e.g., the elongation of the sample was Δ*L* = 4.5–5.5 mm (at *L*_0_ = 330 mm), resulting in relative elongation Δ*L*/*L*_0_ = 1.36%, whereas the elongation Δ*L* = 6 mm (at *L*_0_ = 247 mm) results in relative elongation Δ*L*/*L*_0_ = 2.4%.

NMR and FTIR analyzed the original silk threads before mechanical loading and drawn silk samples. In one type of experiments, constant load was applied to the silk samples on the air at room temperature. Other experiments were carried out at cycling loading, i.e., after constant loading, silk thread was released from the load for a while and then loading was repeated (several loading-release cycles). In the third type of experiments, a constant load was applied to the silk samples in water environment. After elongation in water, the drawn silk threads were left in open air at room temperature overnight and then analyzed using NMR and FTIR methods.

### 2.3. Electron Microscopy

**Figure 2:**
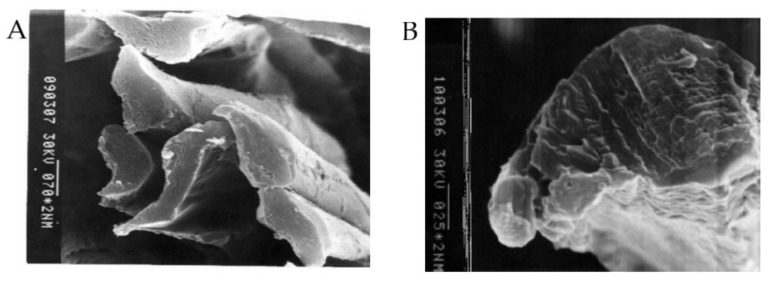
Scanning electron microscopy images characterizing surface of natural silk *Bombyx mori* and cross-section of fibres. (**A**) a group of fibres; (**B**) single thread.

Scanning electron microscopy (SEM) images of silk fibre surface and thread cross-sections were obtained as in an earlier study [41]. A scanning electron microscope S-450 (Hitachi, Tokyo, Japan) with an accelerating voltage for electrons of 30 kV was used. Samples were fixed by narrow strips of a sticky tape on the microscope stage. To visualize the outer surface of polymer thread and cross-section slices of fibres, thin-layer coverage of fibre surface by gold were applied according to previous studies [15,41,42]: gold was sputtered in a vacuum onto the samples using an Eiko IB-3. Figure 2 shows some electronic microphotographs characterizing the surface and cross-section of silk fibres.

### 2.4. FTIR Spectroscopy

FTIR spectra of silk samples were recorded on a Bio-Rad FTS 60 spectrometer (Bio-Rad, Hercules, CA, USA) equipped with a HgCdTe detector [43]. The sample was placed in ATR accessory with a ZnSe crystal for measurement.

**Figure 3:**
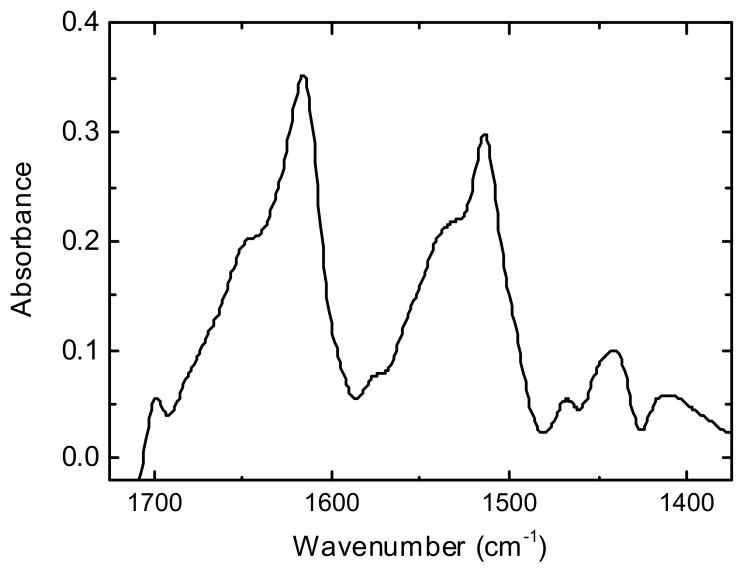
FTIR spectrum of original raw silk fibres of *B. mori* (HL = 0.08 g H_2_O per g dry matter). Amide I and amide II bands have sharp maximums at 1618 cm^−1^ and 1514 cm^−1^, respectively.

All spectra were registered with a resolution of 2 cm^−1^, 32 scans, and wavenumber range from 1400 to 1800 cm^−1^. The empty ATR crystal was used as reference. The FTIR spectra of original silk thread *B. mori* and the silk fibres after treatment (heating/wetting/tensile loading) were recorded. The purpose of these FTIR spectra was to examine how treating *B. mori* silks affects the signals (absorbance) in amide I (1700–1590 cm^−1^) and amide II (1580–1490 cm^−1^) wavenumber regions [43,44,45]. The most significant components of these bands were characterised by peaks of 1695 cm^−1^, 1618 cm^−1^, 1514 cm^−1^, 1650–1660 cm^−1^, and 1533 cm^−1^ as seen in the FTIR spectrum of original silk fibres *Bombyx mori* (Figure 3) [45,46,47]. FTIR examination was not main study of the secondary protein structure of natural silk *B. mori*; therefore, we would rather use the data obtained for a qualitative comparison of silk fibres under different treatment conditions that could not be considered as absolute secondary structure characteristics.

### 2.5. NMR Methods

The NMR studies were performed on an MSL-200 NMR spectrometer (Bruker, Ettlingen, Germany) with a proton operating frequency of 200 MHz (4.68 T). The main purpose of NMR studies was to examine the *T*_1_ and *T*_2_ characteristics in hydrated silk fibres and to clarify orientation anisotropy. The experiments measuring proton relaxation times (*T*_1_, *T*_2_) [48] were carried out using a probe with a 5 mm solenoid coil. The temperature was regulated at 298 K. At studying NMR parameters on temperature, the experiments were realised at a stepping increased temperature from room temperature to 320 K. The 90° pulse length (PL) was between 2.65 and 2.9 µs depending on the sample under the study. Adjustment in PL for each sample was done to achieve maximum signal with this 90° PL or minimum signal (zero) with double 90° pulse. *T*_1_ measurements were made with saturation recovery pulse sequence. This was a way of measuring spin-lattice relaxation times more quickly than with the 180°-*τ*-90° (inversion recovery) sequence [27,31]. The saturation recovery sequence used multiple 90° pulses with delay 20 μs after each 90° pulse in the loop (saturation pulse with PL = 3 μs); number of scans (NS) = 32.

**Figure 4:**
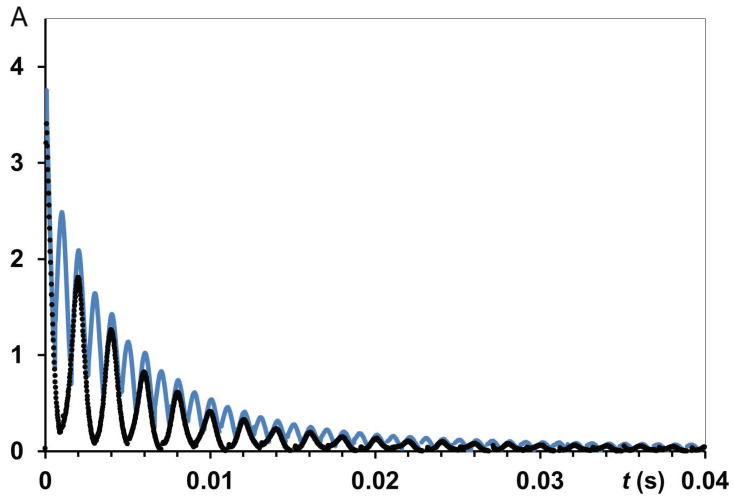
The CPMG pulse train echo intensity as a function of time (*t*, s) measuring from 90° pulse for the silk fibres *B. mori* oriented along *B*_0_; black circles: *τ*_cp_ = 1 ms, echo is formed in every 2 ms; solid blue line: *τ*_cp_ = 0.5 ms, echo is formed in every 1 ms; Dwell time (DW) = 10 μs; NS = 512; HL = 0.60. A is the normalised amplitude of echo signals.

The spin-spin relaxation times of protons (*T*_2_) in silk *B. mori* samples with HL in the range of 0.08–0.15 g H_2_O/g dry matter were measured via the free induction decay (FID) [31,32]. The experimental parameters are SI = 4 k, spectral width (SW) = 1 MHz, with numbers of scans (NS) ranging up to 512. To study wet silk samples (typically HL ~0.33–0.60 g H_2_O/g dry matter), in addition to FID measurements, Carr-Purcell-Meiboom-Gill (CPMG) echo train sequence [31,48,49] was applied, with PL (180°) as a double to PL (90°). For instance, for samples with HL = 0.6 g H_2_O/g dry matter (HL = 0.6), this pulse pair was PL (90°) = 2.85 μs and PL (180°) = 5.71 μs). Interpulse spacing *τ*_cp_ between the pulses in a CPMG sequence was 50 or 100 μs in general *T*_2_ measurements. In some CPMG experiments (using varying *τ*_cp_) *τ*_cp_ varied from 50 μs up to 5 ms. Relaxation time data were analysed using homemade programmes for non-linear multi-exponential fitting measured magnetisation decay within MATLAB software running on a computer. Some examples of CPMG experiments for oriented silk fibres *B. mori* are shown in Figure 4. It can be seen that (at least for *t* ≤ 0.012 s) CPMG decay for *τ*_cp_ = 0.5 ms differed from the one for *τ*_cp_ = 1 ms, i.e., *T*_2_ could be dependent on *τ*_cp_ value.

**Figure 5:**
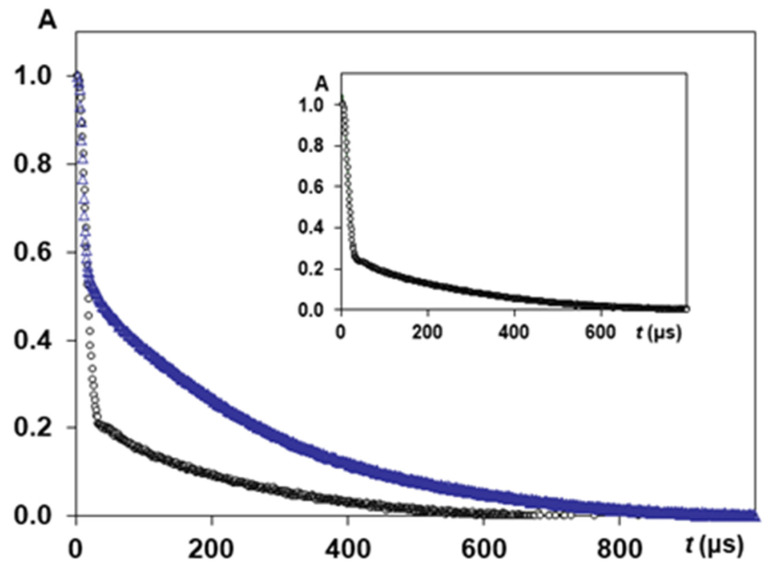
The typical FIDs measured on the silk fibres *B. mori* with perpendicular orientation to static magnetic field *B*_0_; black circles (lower curve): data for dried fibres with low HL = 0.084; blue triangles: FID data for wet fibres with HL = 0.54. The data were normalised per maximum signal in each experiment on dry/wet samples. A—Intensity of NMR signal. (**Insert**): FID data for the silk fibres *B. mori* after tensile loading (89 g) in water environment. NMR experiment was carried out with the perpendicular orientation of stretched silk threads to static magnetic field *B*_0_ at HL = 0.084 and at room temperature. The solid line on the Inset picture is the fitting of experimental FID data to the sum of exponential function and Gaussian-sincexpression: *f*(*t*) = *P*_1_·*exp* [−*a*^2^·*t*^2^/2]·[*sin*(*b*·*t*)/*b*·*t*], where 1/*a* = *T*_2g_—spin-spin relaxation time for fast-relaxing protons.

When NMR pulse sequences were running, relaxation delay was adequate for full magnetization to be reached after each sequence. The free induction decays (FIDs) were initially obtained by registration magnetisation after 90^o^ pulse. The main difficulty in experimental FIDs was receiving correct and reliable points in initial part of the FID curve (before 15 μs). To solve this problem and to increase the reliability of experimental data, we applied the pulse sequence of solid (quadrature) echo. This sequence consisted of two 90° RF pulses separated by a short time gap *τ* = 12 μs (90°_x_-*τ*-90°_y_), slightly exceeding the recovery time interval of the receiver (10 μs). After the 2nd pulse, FID and solid-echo signals were recorded. In a common experiment with FID registration after the first 90° RF, the signal would decay too fast due to rigid dipole-dipole interactions. When applying quadrature echo pulse sequence 90°_x_-*τ*-90°_y_, the signal from the solid protons results in an echo [31,49], i.e., the solid echo pulse sequence refocuses the magnetisation signal. The echo had a roughly Gaussian shape with the center on a time *τ* after 2nd pulse. This provided the possibility of obtaining reliable FID experimental curves with the following treatment to calculate a second moment *M*_2_ [19,48,50]. Thus, FID measurements in solid echo experiment results in more stable and reliable data for NMR signal. Therefore, FID signals in solid echo experiments were studied for all samples. Detailed experimental conditions for this type of experiments were previously reported in [48]. Figure 5 presents examples of experimental FIDs for silk fibres *B. mori*. NMR signal in time domain consists of two clearly discernible parts: a fast decaying component (solid-like) and slower decaying component (liquid-like). The total time domain signal is the sum of the signals from these both parts. The liquid-like magnetisation component could be fitted by exponential function. The solid-like part of the FID was described by Gaussian-since expression: *f*(*t*) *=* *P*_1_·*exp* [−*a*^2^·*t*^2^/2]·[*sin* (*b*·*t*)/*b*·*t*], where *P*_1_ is the signal intensity of the solid-like part of the curve at *t* = 0. Parameters *a* and *b* were used in the expression of the second moment *M*_2_ = *a*^2^ + *b*^2^/3 [15,50,51]. Intramolecular contributions to the second moment were calculated with *b^2^*/*3* [50,51,52]. In an earlier publication on NMR, studying silk fibres *B. mori* with HL = 0.07, it was found that the intramolecular part of the second moment contributes 3.74 × 10^9^ s^−2^, and is determined by the nuclei interaction inside rotating groups. At isotropic rotation, the intramolecular part of second moment should be towards zero. Therefore, for natural silk, this non-zero intramolecular part indicates the anisotropic properties of water molecules in biopolymer [50].

## 3. Results

### 3.1. FTIR Spectroscopy

**Figure 6:**
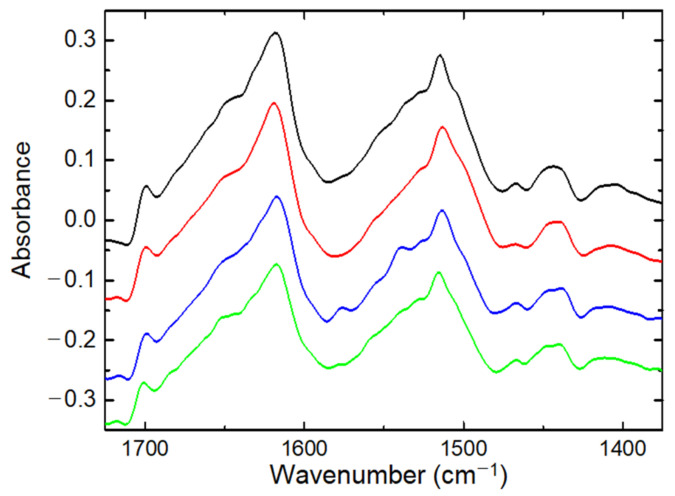
FTIR spectra of silk *B. mori* samples; from top to bottom: (black) original silk *B. mori* cocoon (HL = 0.08); (red) silk *B. mori* cocoon after heating); (blue) silk thread *B. mori* after tensile loading in water (before measurement, the sample was dried in air to the same HL as it was in untreated silk fibre *B. mori*, Figure 3); (green) silk fibres *B. mori* after 7 cycles of tensile loading-release in open air. All measurements were conducted at room temperature.

Figure 6 (black, red curves) shows the FTIR spectra for the silk *B. mori* cocoon before and after heating. This also provides some examples of FTIR spectra for threads after tensile loading in open-air and wet (water) environments. In the silk cocoon of the silkworm *Bombyx mori*, each fibre is composed of two fibroins, coated by a layer of sericin. A comparison of the FTIR findings for the silk cocoon sample with the FTIR spectrum of original silk fibres *B. mori* (Figure 3) discovered similar sharp signals in amide I and amide II bands, as follows: amide I band has the peaks of 1618–1620 cm^−1^, 1640–1650 cm^−1^, and 1695 cm^−1^ whereas in amide II band, the peaks of 1514 cm^−1^, and 1530 cm^−1^ are discovered. According to studies with conformation transitions on silk fibroin [9,46,47], two components at around 1620 and 1695 cm^−1^ are characteristic of *β*-sheet conformation. The peak at 1640–1660 cm^−1^ is attributed to random coil and/or silk I structure [9,46], whereas the shoulder in amide II band at 1533 cm^−1^ could be assigned to unordered loops [47].

Some differences between FTIR spectra for silk raw thread and silk cocoon are associated with an abundance of sericin in cocoon samples: for pure sericin, FTIR spectra discovered the wide peak at 1640 cm^−1^ (random coil) in amide I band. In amide II band, sericin was characterised by peaks of 1515 cm^−1^ and 1533 cm^−1^. A special FTIR study about the effect of sericin on refined silk fibres was not provided, as this was outside the tasks of current research on water in the silk fibres *B. mori*. However, FTIR spectra on pure sericin powder found an absence of *β*-sheets (no peaks at 1620 and 1695 cm^−1^). This emphasizes that the presence of this protein as glue in SF fibres would hide the *β*-structure of SF. Heating silk cocoon resulted in FTIR spectra with intensities of 1695 cm^−1^ and 1620 cm^−1^ peaks that were roughly the same (Figure 6) as when they were discovered in untreated samples. However, there was a small shift in the 1620 cm^−1^ peak, decreasing the intensity of the 1650 cm^−1^ peak and indicating a decrease in random coil (or unordered structure) content.

In amide II band of silk cocoon, the peak at 1515 cm^−1^ is similar to the peak at 1515 cm^−1^ in silk thread *B. mori* (Figure 3). Similarly, in the spectrum of the silk raw thread, there is a little shift in tyrosine peak from 1515 cm^−1^ to 1511 cm^−1^ for silk *B. mori* cocoon after heating. The 1515 cm^−1^ peak intensity slightly diminished after heating the silk cocoon. The signals in amide II band in the silk *B. mori* cocoon were similar to those in this wave-number range for the different silk types: the dragline silk of the spider *Nephila edulis, Actias selene* wild silkworm cocoon. The content of unordered loops (peak at 1533 cm^−1^) decreased after heating silk samples of *Actias selene* wild silk cocoon and spider dragline silk of *N. edulis.*

Treatment of original raw silk *B. mori* with heating results in the conversion of the peak at 1645 cm^−1^ into two bumps at 1640 cm^−1^ and 1655 cm^−1^. The shoulder at 1540 cm^−1^ in amide II band shifted to 1530 cm^−1^ after heating raw silk fibres. In the heated silk fibres *B. mori*, a shoulder appeared at 1558 cm^−1^. The peak at 1575 cm^−1^, observable in original untreated silk fibres, was poor/practically disappeared in the FTIR spectra of heated fibres. After heating, the amide II band showed a discernible decrease in the intensity of the peaks at 1530 cm^−1^ and 1550 cm^−1^, supposing a decrease in unloaded loops.

Tensile loading of silk fibre *B. mori* resulted in small shifts in the 1620 cm^−1^ peak and 1515 cm^−1^ band. The peak at 1575 cm^−1^ in original untreated silk fibres disappeared in the FTIR data of fibres after mechanical loading. The intensity of the peak decreased at 1645 cm^−1^. Small changes in *β*-sheet structure (peak at 1695 cm^−1^) were observed in silk at different variants of constant load (length of thread, weight of load and time of exposition). The amide II band is very sensitive to molecular association via H-bonds. The increased hydration (e.g., when silk threads *B. mori* were under mechanical loading in water environment), is reflected by the increase in absorbance in amide II band at 1530–1540 cm^−1^ (Figure 6).

### 3.2. NMR Results

#### 3.2.1. NMR Studying Randomly Oriented Silk Fibres, *D*_2_*O* Exchange and Silk *B. mori* Cocoon

Table 1 shows the *T*_2_ data obtained in solid echo experiments on randomly oriented silk fibres and a silk *B. mori* cocoon (the data were obtained similarly to Figure 5) at HL = 0.11. For comparison, Table 1 also presents NMR data on silk fibres with higher HL and *T*_2_ data from *D*_2_*O* exchange experiments. These data show that, at HL = 0.11, the protons in randomly oriented silk fibres were characterised by two components in a spin-spin relaxation time: *T*_2g_ = 12.4 μs (proton population 82%) and *T*_2e_ = 133 μs (18%). In the silk *B. mori* cocoon, the solid-like component of cocoon protons has *T*_2g_ = 13.4 μs (73%), whereas the mobile component has *T*_2e_ = 167 μs (27%). Thus, in the silk cocoon, the liquid-like proton component shows a higher proton mobility at the same humidity as that in randomly oriented silk raw fibres. In the silk *B. mori* cocoon, globular protein sericin covers the SF threads resulting in a decrease in the apparent population of the solid proton fraction (to 73%) in comparison with the original population 82% for solid protons in silk raw fibres *B. mori*. The experiment with holding silk fibres in *D*_2_*O* resulted in an exchange of some part of SF protons onto deuterium atoms. Due to the plasticization effect, a population of an apparent proton component with short *T*_2g_ = 12.9 μs decreased (after *D*_2_*O* exchange) to 68%. Thus, an increase in the apparent population of *T*_2_ component with mobile protons (from 18% to 27%) occurred. By increasing HL value to HL = 0.32, the population of long (mobile) *T*_2_ component reaches 48% for randomly oriented silk fibres *B. mori*. At increase in HL in the silk cocoon to HL = 0.24 and a decrease in the population of *T*_2g_ components with solid-like protons (to A_2g_ = 55%) was observed. *T*_2e_ components with liquid like protons increased to A_2e_ = 45% (silk cocoon). The *T*_2e_ = 297 μs for mobile proton component in silk *B. mori* cocoon exceeded *T*_2e_ = 277 μs (silk raw fibres, HL = 0.32), even at a smaller HL (silk cocoon at HL = 0.24). This emphasized the difference between SF in silk *B. mori* cocoon (globular protein sericin “glued” SF threads) and SF in raw silk fibres *B. mori*. Thus, due to the presence of sericin, the *T*_2e_ component of mobile protons increased to *T*_2e_ = 297 μs.

In addition to *T*_2_ findings (Table 1), for these silk *B. mori* samples, we found that the second moment is in the range of (6.45–6.9) × 10^9^ s^−2^ and, for the silk *B. mori* cocoon, *M*_2_ is smaller (i.e., about 5 × 10^9^ s^−2^). Some publications (e.g., McKay et al. [50,53,54]) reported the second instance of *M*_2_ as a value that is dependent on HL for various biological samples. The authors considered the experimental second-moment *M*_2exp_ as the combination of two components, where one is the fraction of protons related to the “rigid” component, and the contribution of this rigid component to *M*_2_ was estimated with some assumptions [50,55].

Studies on spin-lattice relaxation (*T*_1_) in randomly oriented raw silk fibres *B. mori* (HL = 0.11) found a main mobile component (water protons, *T*_1_ = 737 ms) with a population of about 96% and a solid-like protons component (*T*_1_ = 2.5 s) with a small population of 4%. In the silk cocoon, the *T*_1_ value for the water component decreased to 616 ms (HL = 0.10). In random raw silk fibres, after *D*_2_*O* exchange, *T*_1_ for water protons was measured as *T*_1_ = 376 ms (HL = 0.11). Upon increasing HL to HL = 0.24 in silk *B. mori* cocoon, *T*_1_ became *T*_1_ = 326 ms, whereas increasing the HL in random silk to HL = 0.32 resulted in *T*_1_ = 323 ms.

At these treatments, the longer *T*_1_ component (1.6–2.5 s, i.e., only 2–3 times longer than the water component) was not clearly defined or not observable due to its relatively small population (in comparison with the increased signal of the water component). Therefore, we tried also to model the *T*_1_ experiment with only one component. This rough estimation was conducted to compare the effect of HL and *D*_2_*O* exchange in different silk *B. mori* samples. Modelling *T*_1_ data with one component showed a similar tendency (decreasing *T*_1_ with increasing HL) as in the case of two exponential fits: *T*_1_ = 768 ms (random silk at HL= 0.11), *T*_1_ = 421 ms (random silk at HL = 0.32), *T*_1_ = 427 ms in random raw silk fibres after *D*_2_*O* exchange (HL= 0.11), *T*_1_ =647 ms in silk *B. mori* cocoon (HL = 0.10), *T*_1_ = 351 ms in silk *B. mori* cocoon (HL = 0.24). The obtained *T*_1_ data were compared with other *T*_1_ data found in publications on different biomaterials at HL = 0.1–0.3 and (with a *T*_1_ link to the correlation times according to dependence *T*_1_ = *f*(*τ*_c_), considered in Ref [55]), which resulted in *τ_c_* = 10^−7^–10^−8^ s as characteristics of water motion. Thus, in the studied HL range, *T*_1_ decreased with decreasing correlation time.

#### 3.2.2. NMR Studying Orientation Anisotropy in Natural Silk *B. mori* Fibres

Table 2 shows the results of *T*_2_ experiments on the silk fibres *B. mori* samples with different orientations (in parallel (0°) and perpendicular (90°)) to static magnetic field *B*_0_ at HL = 0.085, whereas Table 3 shows the NMR findings of studying anisotropy in oriented (0° and 90°) silk fibres *B. mori* at variable HL.

The *T*_2_ data on silk samples with HL = 0.085 summarise several NMR measurements on separate silk samples that were prepared (and measured) in identical conditions (temperature, HL, and same experimental NMR parameters). The anisotropy of multi-component *T*_2_ relaxation in silk fibres *B. mori* (for orientations of 0° and 90°) was discovered. As each physically different silk sample resulted in there being some differences in anisotropy ratio *R*_2e_ (*R*_2g_), we calculated the mean value for the range of samples (6 samples for 90° orientation and 9 samples for 0° orientation). The mean anisotropy index *R*_2e_ = 1.35 was calculated with the data of water (slow relaxing) *T*_2_ component and *R*_2g_ = 1.14 based on the fast-relaxing *T*_2_ components.

Particular calculations on the silk samples with HL = 0.085 resulted in the following values for anisotropy index, e.g., *R*_2e_ = 1.52, 1.35, 1.43, i.e., some variations near the mean value *R*_2e_ = 1.35, whereas the calculation based on fast-relaxing components at 0° and 90° orientations can result in an anisotropic ratio as follows: *R*_2g_ = 1.10, 1.04, 1.16, 1.14. The mean value (Table 2, HL = 0.085) testifies that the anisotropy effect was registered in systematic experiments for many silk samples. This is not an occasional effect, measured in one or two experiments. It confirmed that anisotropy in multi-component *T*_2_ relaxation (for orientations of 0° and 90°) would be registered if the silk samples were prepared with the same HL. From the proton fractional population of the water (slow-relaxing) *T*_2_ component (Table 2 and Table 3), this index is estimated as 1.05 (at HL = 0.085), i.e., population (proton density) did not show a difference if comparing the 0° and 90° orientations of silk fibres. However, the data (at HL > 0.085, e.g., at HL = 0.11) on proton fraction resulted in an anisotropy ratio of 1.44 (using the population of the slow-relaxing component) and an anisotropy ratio of 1.21 (using the population of the fast-relaxing component). The data in Table 3 show some examples of how increasing HL affects *T*_2_ values (at 0° and 90°) and, consequently, the anisotropy index in silk fibres.

As in randomly oriented silks and silk *B. mori* cocoon (Table 1), the *T*_2_ values of water (slow-relaxing) component increased with HL. The example with HL = 0.11 resulted in a larger anisotropy index compared with that obtained for HL = 0.085 silks.

*T*_1_ values for the slow-relaxing component in oriented silks samples (as in randomly oriented silks and silk cocoon) decreased with an increasing HL. To confirm this tendency, we also conducted an alternative treatment of *T*_1_ experiments as mono-exponential fits. According to Table 3, *T*_1me_ decreased for both orientations of silk fibres (0° and 90°) with increasing HL. *T*_1_ experiments on oriented silks *B. mori* (0° and 90°) at HL = 0.11 showed that *T*_1me_ = 745 ms (0°) exceeds *T*_1me_ = 603 ms (90°) by 1.23 times. However, the second moment 6.9 × 10^9^ s^−2^ (0°) was comparable with 6.4 × 10^9^ s^−2^ (90°) when changing the orientation from 0° to 90° (HL = 0.11). At the same time, increasing the HL for silk fibres *B. mori* (with 90° orientation) decreased *M*_2_ from 6.4 × 10^9^ s^−2^ (HL = 0.11) to 4.8 × 10^9^ s^−2^ (HL = 0.42) and *M*_2_ = 4.1 × 10^9^ s^−2^ (HL = 1.03).

#### 3.2.3. NMR Study of Tensile Loading on Orientation Anisotropy in Natural Silk Fibres *B. mori*

**Figure 7:**
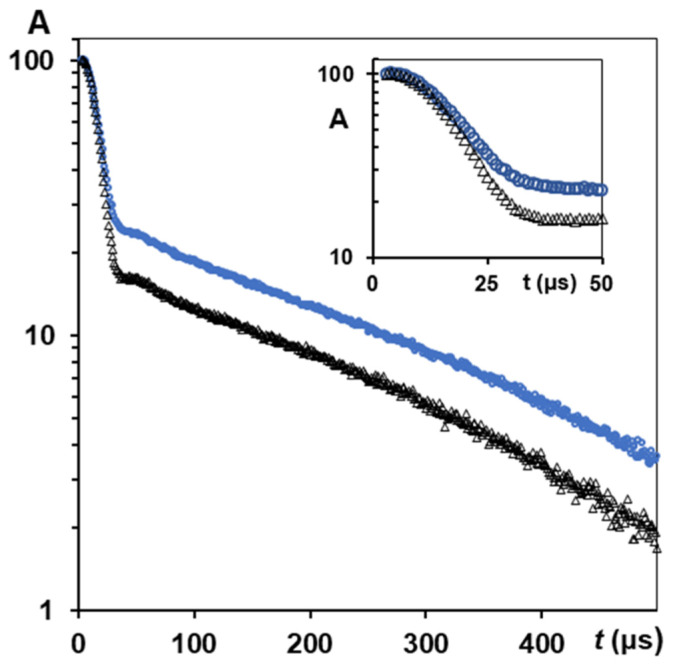
The NMR signals measured (HL = 0.084, orientation 90°) in solid echo experiments on untreated silk fibres *B. mori* (black triangles) and silk fibres *B. mori* after tensile loading (89 g) in water environment (blue circles). The data were normalised per maximum signal in each experiment on these samples. Amplitude (A) of signal (Y-axis) is presented in log-scale (arb.units). Small inset shows initial parts of the decays to better differentiate fast relaxing components.

Figure 7 shows the results of *T*_2_ experiments with a solid echo pulse sequence performed on control silk fibres *B. mori* and silk fibres *B. mori* after mechanical loading in a water environment at measurements with 90° orientation. The population of the slow relaxing component (*T*_2e_ = 214 μs, untreated silk fibres) was 20% (of all measurable protons in the experiment) and increased (27.5%) after tensile loading (*T*_2e_ increased to 232 μs). In order to quantify the anisotropic ratio in the silk fibres after mechanical loading, a series of studies at 0° and 90° orientations was conducted, resulting in the findings presented in Table 4. In order to highlight the effect of mechanical loading more clearly and compare measured NMR parameters with those for untreated silk fibres, we collected these data in Table 5. This presents the *T*_2_ data (0° and 90° orientations, HL = 0.084) for silks *B. mori* (untreated) and for silk fibres measured after the application of mechanical loading. In silk fibres oriented at 90° to static magnetic field *B*_0_, the value *T*_2e_^90^ = 267 μs (after tensile loading) exceeded *T*_2e_^90^ = 212.5 μs (untreated silk fibres) by 1.25 times, whereas, for 0° orientation, the increase in *T*_2e_^0^ (effect of mechanical loading to silk fibres *B. mori*) was 222/157.4 = 1.44.

The anisotropy of *T*_2_ relaxation in silk fibres *B. mori* (0° and 90° orientations) was observed after tensile loading (Table 4). The mean anisotropy ratio was *R*_2e_ = 1.2, as calculated using the data of the slow-relaxing *T*_2_ component, and *R*_2g_ = 1.11 based on the fast-relaxing *T*_2_ component. Some variations in calculated *R*_2e_ (on separate samples) could be observable, e.g., as 1.32, 1.17, 1.30, near to calculated mean value. For fast-relaxing components (at 0° and 90°) measured on separate samples, these variations ranged from *R*_2g_ = 1.02 to *R*_2g_ = 1.42. The mean value (Table 4) shows the anisotropy effect in mechanical loading silk fibres in systematic experiments for four silk samples of each orientation. Thus, if the silk samples were prepared with the same HL, and were tensile loaded under the same conditions, the orientation anisotropy in *T*_2_ relaxation components (0° and 90°) can be measured and quantified. The findings for the second moment showed (at HL = 0.084) that *M*_2_ = 6.1 × 10^9^ s^−2^ (at 0° direction) and *M*_2_ = 5.7 × 10^9^ s^−2^ (at 90° direction), i.e., mechanical loading of the silk threads *B. mori* results in these microstructure characteristics at 0° and 90° orientations.

#### 3.2.4. NMR Study of Temperature Effect on Oriented Natural Silk Fibres *B. mori*

**Figure 8:**
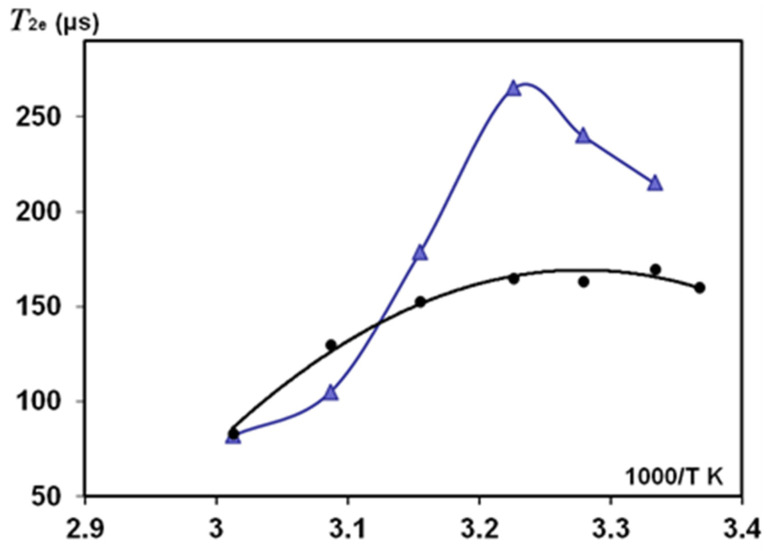
Temperature dependence of the apparent spin-spin relaxation time of slow-relaxing component of water protons (*T*_2e_) in two silk fibres *B. mori* samples (originally HL = 0.083); the samples were slowly heated in the NMR coil and measured after each 5–7° increase in temperature. Solid lines provide guidance for the eye. (1) (Blue triangles) silk fibres with 90° orientation. (2) (Black circles) silk fibres with 0° orientation.

**Figure 9:**
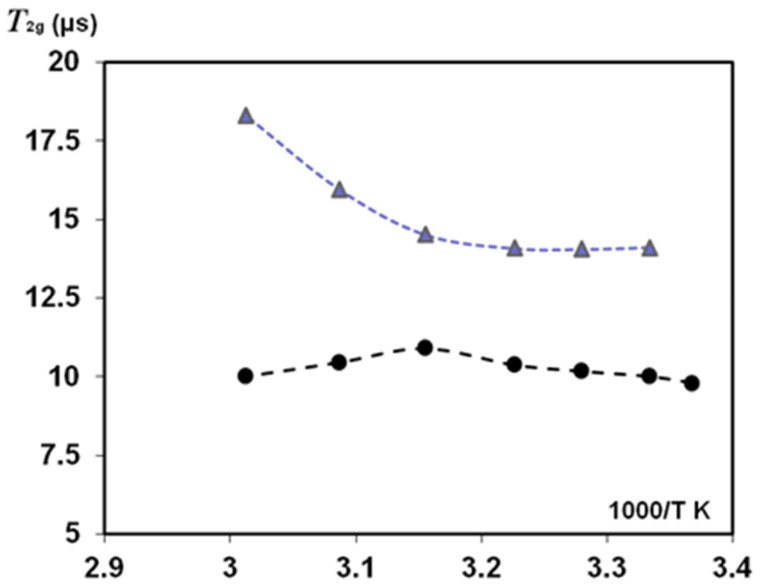
Temperature dependence of the apparent fast-relaxing *T*_2_ component in two silk fibres *B. mori* samples (originally HL = 0.083); the samples were slowly heated in the NMR coil being measured after each 5–7° step. Dash lines provide guidance for the eye. (1) (Blue triangles) silk fibres with 90° orientation; (2) (Black circles) silk fibres with 0° orientation.

Figure 8 and Figure 9 show *T*_2_ findings for oriented silk fibres *B. mori* when these samples (HL = 0.083) were heated in the NMR probe with stepping *T*_2_ measurements at an increasing temperature. At the start of this experiment (T = 297–300 K), slow-relaxing *T*_2_ components (with 0° and 90° orientation) resulted in an anisotropy ratio of 1.28. With increasing temperature, the *T*_2_ component of mobile water protons (at 90° orientation) first increased, as occurred in other porous matrices with a small amount of absorbed water, e.g., in Silica gel and Sephadex gels [56,57]. Those publications found two components in *T*_2_ measurements: the protons with a long relaxation time *T*_2_ (site A) and fast relaxation protons of site *B*. With temperature dependence, the authors [56] investigated the effect of proton exchange between sites *A* and *B* and showed that, for the Silica gel in the temperature range from 283 to 313 K, *T*_2A_ (slow-relaxing component) decreased with increases in temperature, whereas *T*_2B_ was practically non-changeable. The authors of the study on Sephadex gels with absorbed water [57] found that, dependent on the water content of the gel, the changes in *T*_2_ with increasing temperature showed complex behaviour with both maxima and minima in the plots of relaxation rate versus temperature. This was attributed to the interaction of two processes: changes in the contribution of dipolar interactions with temperature, which resulted in an increase in *T*_2_ with increasing temperature, and changes in chemical exchange between protons on the polymer and water, which resulted in a decrease in *T*_2_ with temperature. The interplay of these effects across the temperature range was the cause of the occurrence of maxima and minima in the plots.

These changes reflect the interaction strength between the water molecules and gel matrix by hydration and proton exchange and the differentiation of the flexibility/rigidity of matrix parts. Our results (Figure 8, orientation 90°) showed a similar dependence of *T*_2e_ in the studied temperature range. At the initial increasing in *T*_2e_, and further at *T* ≥ 310 K, *T*_2e_ decreased, showing that the proton exchange could play a role in these *T*_2e_ measurements. *T*_2g_ (for the short-relaxation component) slightly increased in the studied temperature range (90° orientation). However, in the case of 0° orientation, *T*_2g_ is practically permanent (Figure 9).

In the initial temperature range (when temperature begins to exceed the room temperature), *T*_2e_ could increase according to the dipole-dipole mechanism of relaxation. However, this decreased due to the effect of proton exchange (Figure 8). In reality, the mechanisms affecting *T*_2e_ relaxation could be complicated because the silk *B. mori* samples have a very low HL and show orientation anisotropy.

**Figure 10:**
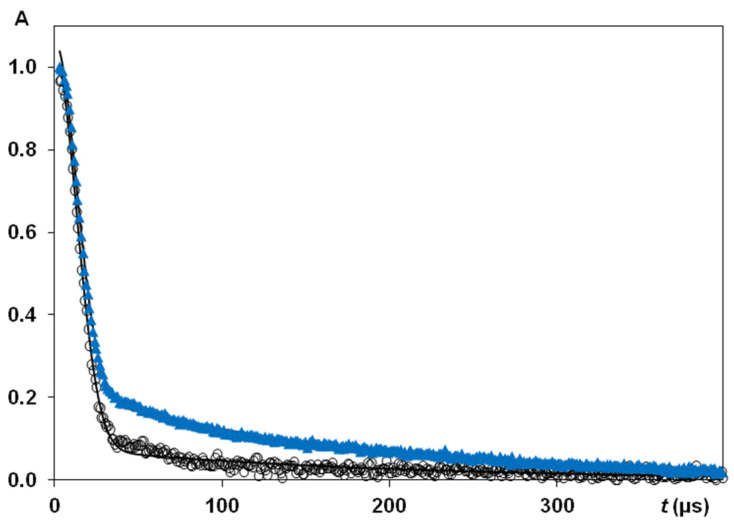
The NMR signals measured (T = 300 K) in solid echo experiments on untreated silk fibres *B. mori* at HL = 0.08 (blue, triangles) and silk fibres *B. mori* after step (5–7°) heating samples in the NMR probe to 350 K (black, circles). After heating, the silk sample returned to 300 K and measured with the same orientation 0°. The data were normalised per maximum signal in each experiment. A (arb. units) is the signal intensity. Solid lines are the fits of the experimental data to the sum of exponential function and Gaussian-sinc expression: *f*(*t*) = *P*_1_·*exp* [−*a*^2^·*t*^2^/2]·[*sin*(*b*·*t*)/*b*·*t*], The details are provided in Section 2.5. NMR Methods.

An anisotropy ratio (that was about 1.3 at the start of the experiment at room temperature) increased to about 1.5 at an initial temperature rise, and this further decreased to 1 as the temperature increased to 320 K. We found that this temperature rise resulted in irreversible changes in silk fibres. The water absorbed inside SF macromolecules was removed by disrupting the original H-bonds without restoring them to their original state after cooling to 300 K (Figure 10). These findings confirm earlier publications on deformation studies of silk fibres *B. mori* at high-temperature (323 K and 373 K) treatment [14,15]. It was shown that σ_max_ in heat-treated silk fibres with a diameter of 21 μm significantly differed (decreased) from the σ_max_ for untreated fibres. This indicates the rearrangement of crystalline regions of the polymer. These regions are sheet structures bound by H-bonds. The regular part of the H chain in crystalline regions is considered to be a sequence of amino acids (-GAGAGS-)_n_. The sheet structure also has serine residues and residual water. As that water is strongly bound to SF, the removal of water by temperature treatment resulted in the destruction of the H-bonds responsible for the sheet-structure sliding upon stretching the fibres (for σ_max_ measurements). Therefore, returning to the same conditions of sample humidity and T = 300 K, water adsorption can develop to slightly different places because the bonds and sheet structure of the solid proton phase irreversibly changed (Figure 10).

#### 3.2.5. CPMG Study of Natural Silk Fibres *B. mori* Spin Exchange

**Figure 11:**
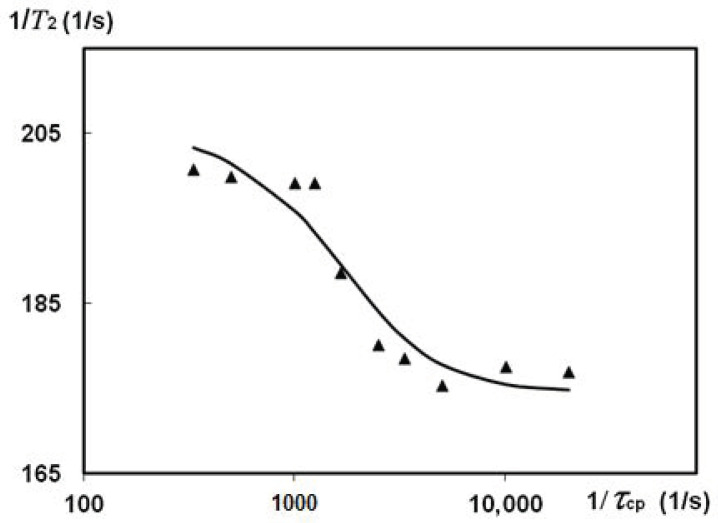
The transverse relaxation rate *R*_2_ measured (T = 300 K) in CPMG experiments on oriented (0° to static magnetic field *B*_0_) silk fibres *B. mori* at HL = 0.60 (black, triangles) varying *τ*_cp_. The data were fitted by the *R*_2_ expression established within two site-exchange theory in the case of fast (*k*_ex_ > Δω) exchange [58,59,60,61,62]. Solid line is the fit of the obtained data to the expression: *R*_2_ = *R*_2_^0^ + (φ/*k*_ex_) [1−tanh(*k*_ex_*τ*_cp_/2)/(*k*_ex_*τ*_cp_/2)] with *R*_2_^0^ = 174.6 s^−1^, *k*_ex_ = 5 793 s^−1^, *φ*/*k*_ex_ =3 2.4, *P*_c_ = 0.8, *φ* = *P*_c_ (1 − *P*_c_)Δω^2^ parameters. *P*_c_ is the fractional population of the protons in site C, whereas a population of site D was described as (1 − *P*_c_). Other details and parameters are provided in the text.

The effect of the proton exchange on *T*_2_ measurement was also investigated at T = 300 K in silk fibres *B. mori* with HL = 0.6. A long exponential decay allowed for CPMG dispersion experiment measuring the spin-spin relaxation rates at different spacing times of *τ*_cp_ between π-pulses. With the CPMG train, we measured *T*_2_ varying *τ*_cp_ in the range from 50 μs to 5 ms. Then, the *R*_2_ findings were expressed as a function of *R*_2_ = *f*(1/*τ*_cp_) according to the accepted model and theory [58,59,60,61]. Figure 11 represents CPMG experiments on silk fibres *B. mori* (HL = 0.6).

An explanation of the experimental results can be provided using the theory of nuclear spin exchange and published findings [58,61,62,63]. The apparent relaxation times are often obscured by the effect of proton transfer. We consider two sites, C and D (these letters are used here instead of A and B, which were introduced in the previous section to analyse the role of proton exchange in temperature experiments), with intrinsic transverse relaxation times *T*_2C_ and *T*_2D_ and different chemical shifts. Then, apparent *T*_2C_* values can be much shorter than *T*_2C_ [56,58]. As a result, an exchange in the µs and ms NMR scale produces an enhanced spin-spin relaxation rate according to: *R*_2_ = 1/*T*_2_ = *R*_2_^0^ + *R*_ex_, where *R*_2_^0^ is the intrinsic spin-spin relaxation rate without distortion by nuclear transfers, and *R*_ex_ is the contribution of dephasing induced by exchange. In the CPMG experiment, pulsing rate (interpulse spacing time) can be varied, from dozens of µs to ms or dozens of ms. If this rate is quite fast in comparison with the lifetime of exchange act, the measurable transverse relaxation time is close to *R*_2_^0^. In [62], the authors conducted simulation studies to describe the exchange process relating CPMG data to an appropriate theory and equations [58,61,63]. They considered variable cases of two and three sites (separated by chemical shift Δω), with slow, fast and intermediate exchanges (i.e., when the exchange rate *k*_ex_ is lower, greater or comparable with Δω). The simulated values covered wide *k*_ex_ ranges (100 to s^−1^) and Δω (250–1500 s^−1^).

We consider that silk fibres *B. mori,* under the studied HL, contain two exchangeable proton pools. On this basis, we tried to use the developed equations to satisfy the findings. Alhough the ‘two phases with exchange’ model is well known, CPMG data on silk *B. mori* were not tested by these theoretical equations [58,61], to fit the *R*_2_ = *f*(1/*τ*_cp_) function according to an accepted model, e.g., for the case of fast exchange. In the vicinity of the SF macromolecules, water molecules have very restricted mobility due to their strong association with preferable locations on the silk. The relaxation rate in this phase is much higher than in another pool with slowly relaxing molecules. A single relaxation rate can be considered as the weighted average of the individual relaxation rates, or as the characteristics of the compartments/environment. Chemical exchange at this timescale could dominate the transverse relaxation as 1/*T*_2_ = *R*_2_^0^ + *R*_ex_ [59]. This has very important consequences for the magnitude of the observed transverse relaxation rate, as measured by the CPMG pulse sequence. In Figure 11, we show that such variations in transverse relaxation rates can be fitted by the Luz and Meiboom equation [58,62]: *R*_2_ = *R*_2_^0^ + (φ/*k*_ex_) [1 − tanh(*k*_ex_*τ*_cp_/2)/(*k*_ex_*τ*_cp_/2)] resulting in *T*_2_^0^ = 5.73 ms and *τ*_ex_ = 4 × 10^−4^ s. The average lifetime of the nuclei between successful exchanges looks reasonable, as it is comparable with the proton exchange time *τ*_ex_ = 1.3 × 10^−4^ s found in [64] when studying the orientation of water molecules in a collagen-water system. Following the simulation approaches [62], we fixed some parameters (e.g., Δω or *P*_C_) and ran a fitting process for the rest of the parameters. This resulted in a deteriorated fitting curve. However, we could obtain a restricted set of parameters that could still fulfil fast exchange restrictions at a diminished Δω compared to that in Figure 11.

## 4. Discussion

The studies of *T*_1_ and *T*_2_ relaxation times in silk fibres *B. mori* with different factors (varying HL, washed in *D*_2_*O*, mechanical loading and heating) and measurement states (oriented *B. mori* threads of 0° and 90°, randomly oriented fibres and SF in cocoon *B. mori*) found different effects of model fitting and anisotropy. The longest *T*_1_ component greatly exceeded the longest *T*_2_ components, and this *T*_1_ value decreased when applying *D*_2_*O* exchange or at an increasing HL (i.e., decreasing correlation times, *τ*_c_). However, this increase in HL resulted in an increase in the longest *T*_2_ value. In the silk cocoon, *T*_1_ for the water component also decreased with the increasing HL and after holding silk fibres in *D*_2_*O*.

A comparison between the silk cocoon and randomly oriented silk raw fibres at the same HL found a higher mobility in water component protons (long *T*_2_ component) in the silk cocoon. The silk *B. mori* cocoon mostly contains fibroin and sericin, as well as waxes and other components. Therefore, the *B. mori* cocoon is a silk composite with a non-woven structure, where the silk (fibroin) core is surrounded by the sericin matrix. The important biological property of sericin is its moisturising properties. As a result, due to the abundance of sericin (this protein envelopes SF fibres with sticky layers) in cocoon silk, the interaction between the cocoon and water should differ from the hydration of silk fibres *B. mori*. The FTIR spectra showed that sericin has no peaks that are responsible for the *β*-structure (comparing the SF FTIR spectra) and can slightly affect these SF signals in the cocoon, especially when the water environment has been applied (e.g., at tensile loading).

In NMR measurements on silk *B. mori* cocoon, we discovered a decreased relative population of the solid proton fraction (up to 73%) in comparison with that in silk raw fibres *B. mori* at the same HL. An increase in HL in silk fibres, or keeping silk fibres in *D*_2_*O*, resulted in plasticization, which decreases the apparent population of *T*_2_ components of solid protons. With the increasing HL in silk cocoons, the relative population of solid protons component decreased, and the population of the water protons increased. In line with this tendency, the spin-spin relaxation time of *T*_2e_ = 297 μs for long component exceeded the *T*_2e_ = 277 μs component in silk raw fibres.

It is possible to explain the *T*_1_ (*T*_2_) data by looking at the different dependences of *T*_1_ and *T*_2_ on correlation times. When looking at the standard expressions for relaxation times linked to spectral functions with resonance frequency and correlation times (presented in [55,56,64]), then function *T*_1_ = *f*(*τ*_c_) has the minimum at ω_0_*τ*_c_ = 0.616. When *τ*_c_ decreases from the minimum in *T*_1,_ relaxation times (both *T*_2_ and *T*_1_) become inversely proportional on *τ*_c_, and they become equal to each other in this *τ*_c_ range (e.g., in liquids). Another *τ*_c_ regime is considered when *τ*_c_ exceeds the correlation time that corresponds to the minimum of the function *T*_1_ = *f*(*τ*_c_). In this range, the *T*_1_ is already proportional to the correlation time, although *T*_2_ is still roughly inversely proportional to *τ*_c_. At a long enough *τ*_c_, the *T*_2_ is limited by the value characterised for the rigid lattice. In this *τ*_c_ regime, the *T*_2_ expression presented in [53,55,56] for *T*_2_ = *f*(*τ*_c_)) does not hold more near these limiting, long *τ*_c_ values for rigid lattice conditions. Thus, the apparent *T*_2_ components for this *τ*_c_ range are much shorter than the *T*_1_ components. In *T*_2_ measurements, the exchange times are relatively long, and this results in clear *T*_2_ components of solid protons and mobile protons of water. These components of *T*_2_ relaxation are orientation-dependent, and the obtained data show how this anisotropy (in oriented 0° and 90° silk fibres *B. mori*) depends on HL.

There was no similar NMR study of hydrated silk fibres *B. mori* in the literature, and we had no opportunity to analyse and compare the data obtained on the natural silks’ orientation anisotropy in NMR relaxation. We could only carry out a comparison with NMR orientation anisotropy data obtained on other heterogeneous biomaterials/tissues (such as collagen fibres and tendons [29,30]). This was mostly conducted to understand the intermolecular interactions that occur when one discovers this orientation anisotropy and to emphasize some issues with these interactions.

In [30], studying the spin-spin relaxation of water in normal and regenerating Achilles tendons at 100 MHz, two *T*_2_ components showed orientation anisotropy for the long *T*_2_ (5.4 ms (0°), 6.21 ms (~55°)) and for the short *T*_2_ component (0.41 ms (0°), 1.43 ms (~55°), and 1.32 ms (90°). The alignment of bound water was caused by the orientation of collagen molecules in normal tendon. Therefore, the dipolar interactions between bound and aligned protons of water define the *T*_2_ relaxation anisotropy, and the strength of these interactions depends on the fibre-to-field angle. The effect of these dipolar interactions is minimal at an orientation when the vector to the proton of the bound water molecule is oriented with a magic angle (~55°) to the static magnetic field. The anisotropy of the molecular arrangement in biomaterials was determined with the aid of angular parameters [29,64,65,66,67,68]. These works showed that the angular factor (1 − 3cos^2^θ) only defines the dipolar splitting of the spectra.

Takamiya et al. [30] showed that the anisotropy index (R_0/90_) in tendon for the proton fractions responsible for the short *T*_2_ component at 0° and 90° measurements was about 3.2. (R_0/55_) = 1.15 is the maximum for the water fraction responsible for the long *T*_2_ component at 0° and 55° [30]. (R_0/90_) will then be even smaller for the long *T*_2_ component (5.4–6.2 ms). Thus, this component does not show obvious anisotropy when tendon is measured with 0° and 90° orientations to the static magnetic field *B*_0_. These authors [30] revealed clear anisotropy in the short *T*_2_ component of the tendon (with *T*_2_ changing of 0.43 to 1.43 ms). In the last example, we highlighted the anisotropy ratio for two mutual orthogonal fibre orientations (0° and 90°). Thus, the anisotropy index (R_0/90_) = 3.2 does mean a comparison between parallel and perpendicular orientation to the static magnetic field *B*_0_ [30]. This provides an opportunity to compare the anisotropy ratio from the literature data with our findings on anisotropy index (R_0/90_) in silk fibres *B. mori* at fibre-to-field angles of 0° and 90°.

In the work of Peto et al. [29], the authors studied orientation anisotropy in *T*_2_-CPMG-measurements (at 20 MHz) on collagen fibres extracted from pig legs when the water content in the samples was 60% in weight. These authors observed four components in *T*_2_-CPMG experiment, but only two of the fastest components (*T*_2_ = 0.85 ms (0°) and *T*_2_ = 4.8 ms (0°), measured at *τ*_cp_ = 100 µs) were dependent on orientation angle, resulting in *T*_2_ = 1.8 ms (55°) and *T*_2_ = 8.2 ms (55°). They associated the protein protons and protons of bound water, respectively, with these *T*_2_ components [29]. Following their data on the fastest two *T*_2_ components, (R_0/55_) is about 2.1 and 1.7, respectively. According to [29], the fastest *T*_2_ component (the protons of macromolecule) changed with the orientation, from *T*_2_ = 0.8 ms (0°) to 1.8 ms (90°). This work showed that the fastest *T*_2_ component is affected by the second fastest *T*_2_ component, which is attributed to the protons of tightly bound water. In that study, two slow *T*_2_ components with *T*_2_ = 16–18 ms and *T*_2_ = 67–80 ms were registered, in addition to the two fast ones discussed above. These slow-relaxing *T*_2_ components did not show orientation anisotropy (no obvious dependence on the angular factor in magnetic field *B*_0_). These two slow components were not discovered by Takamiya et al., in their *T*_2_ study of tendons at 100 MHz [30].

The HL = 0.08–0.40 range was not tested in previous publications on the orientation studies of fibrous materials in a static magnetic field *B*_0_ [29,30,68]. The current study of silk fibres *B. mori* found orientation anisotropy (0° and 90°) at these low HL values when solid echo NMR experiments can be reliably applied to produce *T*_2_ findings with identical parameters and a statistically reproducible manner. We fitted these data with Gaussian-sin*c* and an exponential function for the range of samples: 6 for 90° and 9 for 0° measurements. With these data, the mean anisotropy index (R_0/90_) = 1.35 is for the *T*_2_ component of water bound protons. A maximum effect in (R_0/90_) based on bound water was achieved at HL = 0.08–0.12, i.e., in conditions when the dipolar (*d*–*d*) interaction between tightly bound water and aligned water protons is still effective, and the strength of the *d*–*d* interaction is sensitive to the fibre orientation in the magnetic field. Many different (physically) samples were tested and resulted in this effect. However, this sensitivity to angle variation could become weaker if there is the exchange of bound water protons with the protons of another water environment (e.g., free water) or if the state of solid protons in silk fibre changes with treatment, resulting in weakened *d*–*d* interactions. For example, this can happen in the plasticization effect in a water environment. The mean anisotropy ratio in NMR experiments after tensile loading became 1.21 (using the population of the fast-relaxing component). Table 3 gives several examples of the influence of increasing HL onto *T*_2_ values in silk fibres (at 0° and 90°) and the obtained anisotropy index. In addition to this, the FTIR spectra in amide I band showed some changes in the *β*-sheet structure of silk fibres *B. mori* at tensile loading in water. In the water environment, upon the tensile loading of silk threads, new molecular associations were observed via H-bonds. For these stretched silk threads, FTIR spectra showed the changes in amide II band at 1530–1540 cm^−1^. NMR data also showed the changes in stretched silk threads. Rearrangements of hydrogen bonds were observed in *T*_2_ findings (e.g., *T*_2_ increased; Figure 7). The NMR experiment was performed after the load was removed from the thread, and the thread was returned on the air to the original HL before the tensile loading experiment. It is clear that the plasticization effect resulted in transitions that could change all intermolecular interactions in silk fibres, which possibly changes the strength of dipolar interactions and its angular factor in magnetic fields. In the *T*_2_ measurement with an orientation of 90°, the spin-spin relaxation time also increased. Thus, (R_0/90_) after mechanical loading became (R_0/90_) = 1.2. This decrease suggests that, due to the plasticisation effect, part of the aligned water is in an isotropic state, and the anisotropic effect after additional hydration or tensile loading should be decreased.

In [29,67,68], attempts were made to separately consider (and quantify) these contributions (isotropic and anisotropic parts). The complete effect was found to depend on which relaxation rate increases/decreases faster. The degree of the decreasing *T*_2_ after the treatment of silk thread *B. mori* depends on initial anisotropy ratio (R_0/90_) and strength of the acting factor. The increasing temperature may result in a similar fraction and rearrangement, and consequently, change (R_0/90_). This factor was analysed by considering the temperature-related changes in *T*_2_ (mobile protons and solid protons) components.

The present work studied the influence of increased temperature on *T*_2_ components measured in solid-echo experiments (Figure 8 and Figure 9) and the orientation anisotropy (R_0/90_) in silk fibres *B. mori* at HL = 0.08–0.11. The role of proton exchange has been considered to explain the discovered effects. The decreasing *T*_2_ with increasing temperature discovered in our study on silk *B. mori* is in line with the temperature behaviour of *T*_2_ components in pore materials (silica gel, various gels) [56,57], which these authors consider as the models for tissues and medical materials. The mechanisms developed in the magnetic resonance dispersion studies of gels are often consistent with those in biological tissues [57,69]. The comparison between NMR data (*T*_2_) on silks, published results on model systems and findings in biological materials confirmed the relevance of the acting mechanisms. However, some questions remain and should be noted. On the silk fibres *B. mori*, we showed a role of exchange, considering a two-site exchange model. In water heterogeneous systems, proton exchange between sites separated by a chemical shift is an important process. In the literature, the two-site exchange model is often applied; however, there are also some examples of three-site exchange models [62,67,68]. These examples present the possibility of considering three different fractions of water that slowly exchange water. In addition, each fraction is supposed to contain fast-exchanging sub-fractions. The proton exchange between water molecules and functional groups in the proteins could also be considered. Some researchers noted that exchange models neglect cross-relaxation (CR), i.e., this relaxation can be considered as the result of magnetization transfer at this interface. The fractions with surface relaxation can then act as a relaxation sink for the other fractions. In the NMR studies on natural biomaterials, we detailed these CR effects with simulations and measurements of these cross-relaxation rates, analysing the conditions that contribute to CR in *T*_1_ experiments and diffusion measurements with the stimulated echo pulse sequence [12,16,18,27,70].

## 5. Conclusions

This work developed an approach that can be used to explore orientation anisotropy in natural silk *Bombyx mori* using NMR and FTIR methods. These NMR methods (*T*_1_, *T*_2_ investigation, solid echo, CPMG) were effective when applied to non-oriented silks and oriented (0° and 90°) silk fibres in a magnetic field for some factors (tensile loading, heating, hydration and exchange), enabling an observation of the orientation anisotropy of water molecules in silks. The dipolar interactions that involve the bound water and aligned water protons define *T*_2_ relaxation anisotropy in silk fibres in a static magnetic field.

An analysis of the different factors and mechanisms that can be used to discover anisotropy in the water mobility of hydrated silks at the studied natural and experimental conditions could be further developed in natural polymers and synthetic materials with planned (oriented) properties. These features can be affected by increasing/decreasing the water content and other factors connected with hydration level, temperature regime or mechanical loading changing interactions in silk polymer domains. Water molecules’ ability to penetrate the non-crystalline disordered domains of silks and increase the mobility of polymer chains depends on tensile loading threads and temperature action. The fibroin of silk fibres *B. mori* has a large number of high-oriented parts. These parts are important, especially at a low HL. The obtained data provide new insight into the role of these oriented parts, an analysis of the factors affecting orientation anisotropy in measured NMR relaxation times and an understanding of the reasons and mechanisms of these changes. The research data might increase the understanding of the mechanical properties of silk fibres and find future applications of silks. With the obtained *T*_2_ and R_0/90_ data, we can determine how the differentiation of *T*_2_ components could be realised in different silk materials, and how identification of the anisotropy index could be used to monitor the structural characteristics of varying silk materials. With these results on silk fibres *B. mori*, this paper can assist new NMR studies detailing water interactions in silk-based materials with orientation anisotropy.

## Figures and Tables

**Table 1:** NMR relaxation times *(T*_2_) of protons in randomly oriented silk fibres and in *Bombyx mori* cocoon. Effect of *D*_2_*O* on exchangeable protons in silk fibres *B. mori*.

	*T*_2g_ (μs)	A_2g_ (%)	*T*_2e_ (μs)	A_2e_ (%)
Randomly oriented fibres, HL = 0.11	12.4	82	133	18
Randomly oriented fibres, HL = 0.32	8.5	52	277	48
Fibres after *D*_2_*O* exchange, HL = 0.11	12.9	68	193	32
Silk *B. mori* cocoon, HL = 0.10	13.4	73	167	27
Silk *B. mori* cocoon, HL = 0.24	16.4	55	297	45

**Table 2:** *T*_2_ data on the silk fibres’ *B. mori* samples with different orientations (0° and 90°) to basic magnetic field *B*_0_ at HL = 0.085. *T*_2_ values are presented as means for N experiments (N separate silk samples prepared and measured) with standard deviation (SD). For 90° orientation of silk fibres, N = 6, whereas, for 0° orientation, N = 9. *R*_2e_ = *T*_2e_^90^/*T*_2e_^0^ is the degree of anisotropy/anisotropy ratio based on slow-relaxing *T*_2_ component (water protons), and *R*_2g_ = *T*_2g_^90^/*T*_2g_^0^ is the degree of anisotropy/anisotropy ratio, calculated with fast-relaxing *T*_2_ component (solid protons) data.

	N	*T*_2e_ (μs)	SD	A_2e_ (%)	*R_2e_ = 1.35*	*T*_2g_ (μs)	SD	A_2g_ (%)	*R*_2g_ = 1.14
Orientation 90°	6	212.5	7.4	20.2		14.4	0.9	79.7	
Orientation 0°	9	157.4	14.3	19.2		12.6	1.1	80.8	

**Table 3:** *T*_2_ and *T*_1_ data on the silk fibres’ *B. mori* samples with parallel and perpendicular orientations to *B*_0_ at different HL = 0.085; 0.11; 0.35; 0.42; 1.03. The result with mono-exponential fitting is marked as *T*_1me_.

	HL	*T*_2e_ (μs)	A_2e_ (%)	*T*_2g_ (μs)	A_2g_ (%)	*T*_1e_ (ms)	A_1e_ (%)	*T*_1s_ (s)	A_1e_ (%)	*M*_2_(× 10^9^ s^−2^)	*T*_1me_ (ms)
Orientation 0°	0.085	150.3	20	12.8	80	--	--	--	--	--	--
Orientation 0°	0.11	162.7	18	11.7	81	802	95	1.29	5	6.9	745
Orientation 0°	0.35	429.0	55	9.5	45	--	--	--	--	6.1	451
Orientation 90°	0.085	215.2	19	14.1	81	--	--	--	--	--	--
Orientation 90°	0.11	260.1	26	12.1	74	464	74	1.26	25	6.4	603
Orientation 90°	0.42	557.5	50	10.3	50	362	82	0.93	16	4.74	314
Orientation 90°	1.03	597.0	58	11.0	41	326	--	--	--	4.12	326

**Table 4:** *T*_2_ data on the silk fibres *B. mori* samples (at HL = 0.084) measured at 0° and 90° orientation. *T*_2_ values are presented as means for N experiments with standard deviation (SD). *R*_2e_ = *T*_2e_^90^/*T*_2e_^0^ is degree of anisotropy based on slow-relaxing *T*_2_ component (water protons), and *R*_2g_ = *T*_2g_^90^/*T*_2g_^0^ is degree of anisotropy calculated with fast-relaxing *T*_2_ component (solid protons) data. (*L*_1_–*L*_2_)/*L*_1_ = 1.7–2.4%.

	N	*T*_2e_ (μs)	SD	A_2e_ (%)	*R*_2e_ = 1.2	*T*_2g_ (μs)	SD	A_2g_ (%)	*R*_2g_ = 1.11	*M*_2_ (× 10^9^ s^−2^)	SD
Orientation 90°	4	267.0	28.1	27.5		13.4	0.8	72.5		5.7	0.1
Orientation 0°	4	222.1	7.4	29.2		12.1	2.1	70.7		6.1	0.1

**Table 5:** NMR relaxation times (*T*_2_) of protons in oriented (0° and 90°) silk fibres (untreated) and in silk fibres *Bombyx mori* after tensile loading. HL = 0.084. The data for silk with mechanical treatment are marked with (*).

	*T*_2e_ (μs)	A_2e_ (%)	*T*_2g_ (μs)	A_2g_ (%)	*T*_2e_* (μs)	A_2e_* (%)	*T*_2g_* (μs)	A_2g_* (%)
Oriented fibres, 90°	212.5	20.2	14.4	79.7	267.0	27.5	13.4	72.5
Oriented fibres, 0°	157.4	19.2	12.6	80.8	222.0	29.2	12.1	70.7

## Data Availability

The data in the figures are available upon request from: (PC: V.V. Rodin). The software used for simulations and analysis is available upon request.

## Dedication

The paper is dedicated to Professor Norbert Müller to mark his contribution to NMR.
